# Primary stroke prevention for sickle cell disease in north-east Italy: the role of ethnic issues in establishing a Transcranial Doppler screening program

**DOI:** 10.1186/1824-7288-35-15

**Published:** 2009-06-22

**Authors:** Raffaella Colombatti, Giorgio Meneghetti, Mario Ermani, Marta Pierobon, Laura Sainati

**Affiliations:** 1 Clinic of Pediatric Haemathology Oncology, Department of Pediatrics, University of Padova, Italy; 2 Clinic of Neurology, Department of Neurological Sciences, University of Padova, Italy

## Abstract

**Background:**

Stroke is a serious complication of sickle cell disease (SCD) in children. Transcranic Doppler (TCD) is a well-established predictor of future cerebrovascular symptoms: a blood flow velocity >200 cm/sec in the Middle Cerebral Artery (MCA) correlates with a high risk of stroke in cohorts of African-american HbS/HbS patients. In North-East Italy the recent increase in SCD patients is mainly due to immigration from Africa. A comprehensive care program for children with SCD was established in our Center since 2004, but a wide and routine screening for Primary stroke prevention needs to be developed.

**Methods:**

In order to verify the feasibility of TCD and Transcranial color coded Sonography (TCCS) screening in our setting and the applicability of international reference values of blood velocities to our population of African immigrants with HbS/HbS SCD, we performed TCD and TCCD in 12 HbS/HbS African children and two groups of age-matched controls of Caucasian and African origin respectively. TCD and TCCS were performed on the same day of the scheduled routine hematologic visit after parental education.

**Results:**

All parents accepted to perform the sonography to their children. TCD and TCCD were performed in all patients and an adequate temporal window could be obtained in all of them. Pulsatility index and depth values in both the MCA and the Basilar Artery (BA) were similar at TCD and TCCS evaluation in the three groups while time-average maximum velocities (TAMM), peak systolic velocity and diastolic velocity in the MCA and BA were higher in the patients' group on both TCD and TCCS evaluation. African and Caucasian healthy controls had similar lower values.

**Conclusion:**

Our preliminary data set the base to further evaluate the implementation of a primary stroke prevention program in our setting of HbS/HbS African immigrants and HbS/beta thalassemia Italians. Parental education-preferably in the native language- on stroke risk and prevention in SCD increases compliance and should be a necessary part of the program. Ethnic background does not seem to influence TCD velocity and internationally accepted reference values already validated in African-American SCD pediatric patients can be used, but long prospective trials are needed to verify their efficacy in defining stroke risk in our setting.

## Background

Inherited hemoglobin disorders are an increasing global health problem[1]. Italy is a natural reservoir of Sickle Cell Disease (SCD) due to the high frequency of the S gene in the Sicilian population (2–13%) [2] and to the high frequency of beta thalassemia mutations in the Italian population (6–11%)[3]. HbS/HbS and HbS/βthalassemia forms of SCD are therefore already present in Italy. Globalization, with its population movements, brought to the Northern Regions of Italy immigrants from areas where other SCD variants are even more frequent (HbS/HbS with different aplotypes or HbS/HbC) [1]. This led to an increase of SCD patients, mainly African immigrants [4] and raises the need to organize a network of care tailored to the specific needs of our population of SCD patients.

In fact, SCD shows extreme phenotypic variability among individuals and among populations [1,2,5]. Both environmental and genetic factors are likely to contribute to most manifestations of HbS/HbS SCD disease that develops with different patterns in various ethnic groups and individuals [5,6].

Stroke is a frequent complication of SCD. Approximately 10% of African-american patients have a clinical stroke before 20 years of age [7] and another 22% have a silent infarction on magnetic resonance imaging [8]. Stroke has also been described in Mediterranean [6,9] and African SCD patients [10-13] even though with lesser frequencies (4.1% and 6.7% respectively).

Screening programs for stroke prevention in SCD children have been implemented in the United States and in some European countries where comprehensive services for SCD patients are part of routine care [14-17] using Transcranial Doppler (TCD). TCD can measure flow velocities in the large intracranial arteries. The narrowing of these arteries, which leads to cerebral infarction is characterized by an increased velocity of flow. Long term prospective studies have demonstrated the validity of TCD in identifying children at risk of stroke due to increased cerebral velocities[14]. These studies have also demonstrated that prevention of the first stroke (primary prevention) can be done with success with chronic transfusion in children at risk for stroke according to abnormal blood flow TCD velocities [15]. Although TCD cannot predict all strokes, TCD and TCCS offer an opportunity to apply an effective therapy (chronic transfusion) for patients in the risk group [7,14,15].

TCD/TCCS Reference peak-flow velocity values defining normal, conditional and at-risk patients (TAMM <170, 170–200, >200 cm/sec respectively) have been defined through long-term prospective studies performed in African-american patients with HbS/HbS or HbS/HbC disease in the United States [7,14]. The same reference values have also been used in Caucasian patients with HbS/βthalassemia in Greece demonstrating an equal validity in predicting stroke[16]. But TCD or TCCS studies are not yet performed in African countries and normal reference values or baseline data for African SCD patients or for healthy Africans are therefore not yet available. The stroke predictive power of TCD in African populations has therefore not been demonstrated.

In Italy a National Comprehensive Program for the care and treatment for SCD is still missing and a routine screening with TCD able to reach the majority of SCD pediatric patients has still to be implemented. Italian national reported data on TCD values are mainly from Caucasian adult patients during acute stroke or from the healthy adult female population [18,19]. A survey on the healthy pediatric Italian population or the healthy first generation of African immigrant children is missing.

One of the major concerns in TCD/TCCS screening programs in US and UK is the limited access that patients and family have to routine TCD/TCCS exams. Several studies have in fact outlined that even though TCD screening is included in the American National Institute of Health (NIH) guidelines [20] and British National Health System (NHS) [21] guidelines of standard health maintenance for SCD children, wide application of this procedure is uncommon [21-24]. Overburdening children and families with too many appointments and not including TCD/TCCS in routine hematological visits are one of the causative factors of non adherence to TCD screening. Others include poor understanding of TCD/TCCS usefulness to prevent serious complications by the parents. These issues are of particular concern when dealing with a population of first-generation immigrants – as the one that mainly constitutes the group of SCD pediatric patients in Italy- and must be taken into account in the design of a primary stroke prevention program in our setting.

In order to set up a Primary Stroke Prevention Program in our region, we performed a pilot study to determine the feasibility of TCD and TCCS in a group of African children with SCD routinely followed at the Clinic of Pediatric Hematology-Oncology of the University of Padova.

With the objective of increasing acceptance of TCD/TCCS screening and compliance to follow-up we stressed the educational part, performed in different languages

With the aim of verifying if internationally accepted reference values could also apply to our population and could be used as reference screening values, we compared cerebral velocities, pulsatility indexes and depth of the patient's group to those of two groups of healthy age-matched controls of Caucasian and African origin respectively.

## Materials and methods

### Patients enrolment

TCD and TCCS screening were proposed to the parents of African children with SCD routinely followed at the Clinic of Pediatric Hematology-Oncology of the University of Padova. After explaining the neurological complications of SCD and the possibility to identify children at risk of stroke with TCD/TCCS, the opportunity to perform the screening during one of the scheduled routine hematology visits was offered. To be sure of proper understanding, explanations to parents were performed not only in Italian, but also in English or French, according to the need.

Age-matched controls were recruited among healthy Caucasian and African children known by the physicians.

### TCD-TCCS

TCD and TCCS were performed on the same day of a scheduled hematology visit and in the same hospital complex were the Pediatric Hematology-Oncology Clinic is located.

TCD and TCCS were performed using a 2 MHz pulsed Doppler ultrasonograph (EME TCD 2000/S) and a ATL HDI 3000/S Echo Doppler system respectively. Patients were not sedated during the examination.

We have measured peak systolic blood flow velocities (PSV), end diastolic blood flow velocities (EDV), time-averaged mean velocity of maximum blood flow (TAMM) and mean blood flow velocities (MV), at the level of the Basilar Artery (BA) and of the Middle Cerebral Artery (MCA) on both sides of the brain.

We used the Stroke Prevention Trial in Sickle Cell Anemia Study (STOP) criteria [7,14,15], which are based on the intracranial velocities (TAMM) measured in the MCAs, by the TCD method, to assign stroke risk as low (TAMM < 170 cm/sec), conditional (TAMM ≥ 170 – 199 cm/sec), or high (TAMM ≥ 200 cm/sec).

In our study we decided to assess also the blood flow velocities of the BA as we wanted to explore the intracranial hemodynamics of the Vertebro Basilar circulation in SCD (an arterial territory not evaluated in the STOP Trial). The intracranial blood flow velocities can have a wide variations in different normal subjects depending on several factors (age, angle correction, diameter of the vessel).

### Statistical Analyses

The Kolmogorov-Smirnov test was used to preliminary check the normality of the variables. The One-Way Analysis of Variance was then performed. The significance level was set at p < 0.05.

## Results

Thirty-six children were enrolled: group A with 12 SCD HbS/HbS African patients, Group B with 12 healthy African age-matched controls, Group C with 12 healthy Caucasian age-matched controls. Each group had 8 Females and 4 Males. Mean age was 4.51 ± 2.87 years (range: 1.06–9.86), 5.75 ± 3.38 (range:1.21–12.55), 6.84 ± 3.6 (range: 1.41–12.07) for GROUP A, B and C respectively with no significantly statistical difference (p = 0.55).

TCD and TCCS were performed in all patients and an adequate temporal window could be obtained in all of them.

**Figure 1:**
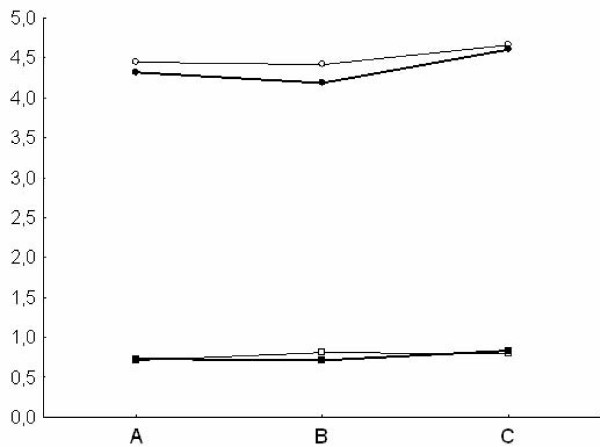
**Middle Cerebral Artery: Mean of Depth (circle) and Pulsatility index (square) in TCD (narrow line) and TCCS (thick line) in the three groups of patients A, B, C**.

**Figure 2:**
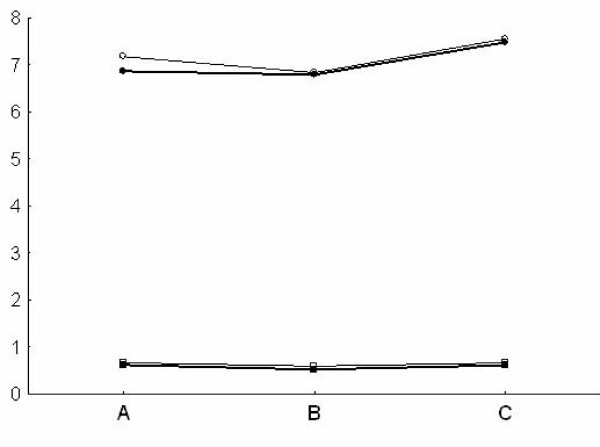
**Basilar Artery: Mean of Depth (circle) and Pulsatility index (square) in TCD (narrow line) and TCCS (thick line) in the three groups of patients A, B, C**.

**Figure 3:**
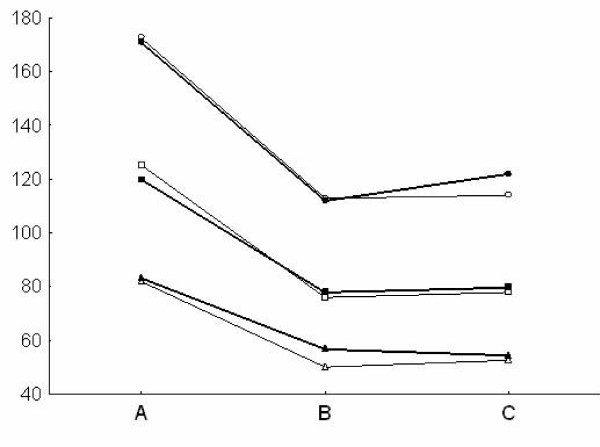
**Middle Cerebral Artery: Mean of PSV (circle), TAMM (square) EDV (triangle) in TCD (narrow line) e TCCS (thick line) in the three groups of patients A, B, C**.

**Figure 4:**
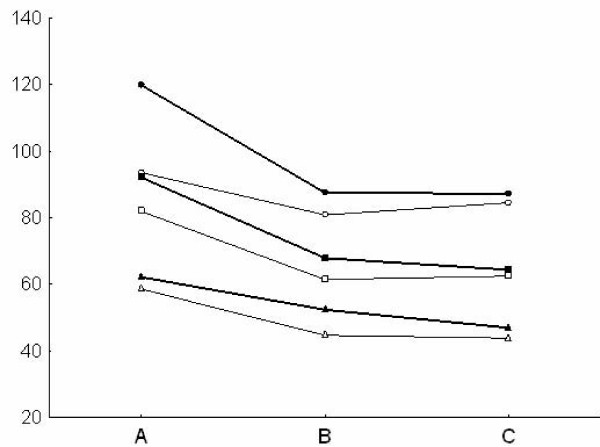
**Basilar Artery: Mean of PSV (circle), TAMM (square) EDV (triangle) in TCD (narrow line) e TCCS (thick line) in the three groups of patients A, B, C**.

Pulsatility index and depth values in both the MCA and the BA (Fig 1, 2) were similar at TCD and TCCS evaluation in the three groups. TAMM, PSV and EDV in the MCA and BA (Fig. 3, 4) were significantly higher in group A patients on both TCD and TCCS evaluation (p = 0.0001 and p = 0286, respectively). No difference was observed between the right and left side. TCCS values were slightly higher that TCD values, with no statistical difference.

Three GROUP A patients had conditional velocities, the remaining being normal. In our population none had abnormal velocity, even though the overall mean velocities were higher than control ones.

## Discussion

Evaluating immigrant children with SCD using TCD and TCCS for primary stroke prevention in our setting is feasible, even if neurosonology examinations are not yet routinely performed for children or adolescents. All parents accepted to perform TCD and TCCS and both exams were feasible in all children. Performing TCD and TCCS during a routine visit at the Hematology Clinic without over burning families with other appointments, might have helped in assuring a positive answer and in assuring compliance for successive controls as happened in other countries [21-24]. The education of parents on neurological complications of SCD and the benefits of screening, as well as the reassurance on the lack of risk of the TCD procedure, emerges as a fundamental component of a successful stroke prevention program in our setting. Performing education in a language that parents can understand, feeling free to expose doubts and ask questions without the linguistic barriers that immigrant parents and patients generally experience, is surely a simple way to increase adherence and participation.

Greater variability of TCCS compared to TCD in our population correlates with previous reports [25]. TCD was easily performed in pediatric patients than TCCS due to the less time needed to perform the exam.

Extreme phenotypic variability is a landmark of SCD [1,6]. Various reports describe different clinical patterns in different populations, according to haplotype of the βglobin genes and related genes [26]. Various reports indicate also cerebrovascular complications of SCD as happening with different prevalence in different populations. Even though excess stroke risk exists in blacks [27,28], it has been postulated that stroke occurs with less frequency in African SCD children compared to Caucasians or African-americans [10,11,29], questioning the need to perform regular screening in these populations. A 11% prevalence of stroke has been observed in the American Cooperative Study of Sickle Cell Disease [7,8] while a 6.7% prevalence of clinically evident stroke has been reported in first generation SCD African immigrants living in France [30]. In the French cohort of homozygous HbS/HbS african children, abnormal TCD has been reported in 9.6% of cases [30,31], while abnormal TCD was present in 9.5% to 12% of the American cohorts [14,15].

In our study, Caucasian healthy and African healthy children had similar low values of all parameters measured on TCD and TCCS. SCD African patients had higher mean values of velocities, but pulsatility and depth were similar to healthy controls, both white and black. These data suggest that ethnic background does not influence baseline normal values of pulsatility, depth and velocities parameters and that SCD is the only factor influencing and determining velocities increase. Pulsatility index, indicating vascular resistance, does not seem to be influenced by SCD being the same in the three groups of children.

In our group of patients, at the time of the analysis, none presented abnormal velocities even thought mean velocities were higher that in controls. Three children (25%), however, had conditional values. The lower Mean age in our group compared to the one of the French cohort (mean 10.1 ± 5.8 years and median 9.3 years) [30,31] could partially be taken as an explanation of this data. Longer follow up and a larger population is needed to precisely define the risk of stoke and the percentage of abnormal TCD in African children with SCD. The three children that had conditional velocities (170–200 cm/sec) at initial evaluation, developed abnormal high velocities (>200 cm/sec) after six months and had to be placed on a chronic transfusion program; one child developed ischemic stroke while waiting for the first transfusion. These preliminary data suggest that a primary stroke prevention program for our population is necessary and set the base to further evaluate the implementation of such a program in our setting of HbS/HbS African immigrants and HbS/beta thalassemia Italians.

With appropriate training and coordination between Pediatric Sickle Cell Groups and Neurosonology Services, the costs of the screening could be very limited. The Adult Neurosonology Services that are already developed across Italy for the evaluation of Adult Stroke could use the equipment and the personnel already available in the Adult Stroke Team to perform TCD screening in pediatric sickle cell patients. For the present being, the small number of pediatric sickle cell patients is not likely to over burn the neurosonology services and to raise the need of adjunctive pediatric neurosonology services

Investigating multiple cerebral vascular districts, besides the MCA, could aid in defining the ones that can be easily monitored and that could better predict future stroke.

## Conclusion

Evaluation of blood flow in the MCAs and in the BA through two different intracranial ultrasonographic techniques is feasible in pediatric SCD African immigrants. A routine primary stroke prevention program should be implemented as part of comprehensive standard health evaluation for SCD children in our setting and parent education-possibly in the native language-should be a necessary part of the program.

The definitive validity in our setting of the internationally accepted reference cut-off values already in use in other Caucasian and African-american SCD pediatric patients should be confirmed in a wide prospective study.

## Competing interests

The authors declare that they have no competing interests.

## Authors' contributions

All the authors contributed equally to the research and to the manuscript.

RC designed the study, supervised data acquisition and interpretation; wrote the manuscript and gave the final approval. GM designed the study, performed the analysis supervising data interpretation and revised the manuscript. ME performed data analysis, participated in data interpretation and revised the manuscript. MP designed the study, collected the data and revised the manuscript. LS designed the study, supervised data acquisition and interpretation; revised the manuscript and gave the final approval
